# Analysis of the role and mechanism of EGCG in septic cardiomyopathy based on network pharmacology

**DOI:** 10.7717/peerj.12994

**Published:** 2022-03-09

**Authors:** Ji Wu, Zhenhua Wang, Shanling Xu, Yang Fu, Yi Gao, Zuxiang Wu, Yun Yu, Yougen Yuan, Lin Zhou, Ping Li

**Affiliations:** 1 Department of Cardiovascular, The Second Affiliated Hospital of Nanchang University, Nan Chang, China; 2 Department of Cardiovascular, Medicine, Fuzhou First People’s Hospital, Fu Zhou, China; 3 Department of Cardiovascular, The Three Affiliated Hospital of Nanchang University, Nan Chang, China

**Keywords:** EGCG, Septic cardiomyopathy, Network pharmacology, Inflammation, Apoptosis

## Abstract

**Background:**

Septic cardiomyopathy (SC) is a common complication of sepsis that leads to an increase in mortality. The pathogenesis of septic cardiomyopathy is unclear, and there is currently no effective treatment. EGCG (epigallocatechin gallate) is a polyphenol that has anti-inflammatory, antiapoptotic, and antioxidative stress effects. However, the role of EGCG in septic cardiomyopathy is unknown.

**Methods:**

Network pharmacology was used to predict the potential targets and molecular mechanisms of EGCG in the treatment of septic cardiomyopathy, including the construction and analysis of protein-protein interaction (PPI) network, gene ontology (GO), and Kyoto Encyclopedia of Genes and Genomes (KEGG) pathway analysis and molecular docking. The mouse model of septic cardiomyopathy was established after intraperitoneal injection of LPS (lipopolysaccharide). The myocardial protective effect of EGCG on septic mice is observed by cardiac ultrasound and HE staining. RT-PCR is used to verify the expression level of the EGCG target in the septic cardiomyopathy mouse model.

**Results:**

A total of 128 anti-SC potential targets of EGCGareselected for analysis. The GO enrichment analysis and KEGG pathway analysis results indicated that the anti-SC targets of EGCG mainly participate in inflammatory and apoptosis processes. Molecular docking results suggest that EGCG has a high affinity for the crystal structure of six targets (IL-6 (interleukin-6), TNF (tumor necrosis factor), Caspase3, MAPK3 (Mitogen-activated protein kinase 3), AKT1, and VEGFA (vascular endothelial growth factor)), and the experimental verification result showed levated expression of these 6 hub targets in the LPS group, but there is an obvious decrease in expression in the LPS + EGCG group. The functional and morphological changes found by echocardiography and HE staining show that EGCG can effectively improve the cardiac function that is reduced by LPS.

**Conclusion:**

Our results reveal that EGCG may be a potentially effective drug to improve septic cardiomyopathy. The potential mechanism by which EGCG improves myocardial injury in septic cardiomyopathy is through anti-inflammatory and anti-apoptotic effects. The anti-inflammatory and anti-apoptotic effects of EGCG occur not only through direct binding to six target proteins (IL-6,TNF-α, Caspase3, MAPK3, AKT1, and VEGFA) but also by reducing their expression.

## Introduction

Septic cardiomyopathy is a common complication of sepsis and an important factor in the high mortality of sepsis. The incidence of septic cardiomyopathy in sepsis is as high as 10–70% (Sarah et al., 2018). To date, the pathogenesis of septic cardiomyopathy is still not fully understood. Many mechanisms are involved in the occurrence and development of septic cardiomyopathies, such as inflammatory reactions, mitochondrial dysfunction, oxidative stress, apoptosis and abnormal calcium regulation (Martin et al., 2019). At present, the main methods for the treatment of septic cardiomyopathy are as follows: vasoconstrictor drugs, fluid resuscitation, cardiotonic drugs, heart rate control, mechanical support and other emerging treatments (Heureux et al., 2020). However, there is currently no drug fully effective at treating septic cardiomyopathy.

(-)-Epigallocatechin-3-gallate (EGCG) belongs to the family of catechins, is a secondary metabolite found in tea and is considered a potential substitute for synthetic food additives due to its antioxidant and antibacterial activities (Nikoo, Regenstein & Ahmadi Gavlighi, 2018). EGCG exhibits good performance in the study of some inflammation-related diseases  (Li et al., 2021b; Huang et al., 2021; Xie et al., 2020; Kar et al., 2019). Recently, it has been reported that EGCG may participate in creating a protective effect against COVID-19 through anti-inflammatory and antioxidant effects (Zhang et al., 2021b). Ma et al. (2021) found that EGCG could protect against liver and intestinal tract injury induced by LPS by stabilizing intestinal flora. In addition, EGCG can also protect against LPS-induced neurological damage (Cheng et al., 2021) and lung injury (Wang, Fan & Zhang, 2019). However, the role of EGCG in septic cardiomyopathy is unknown.

A network pharmacology-based approach has previously proven successful in revealing treatable targets and mechanisms from bioinformatics assays (Kibble et al., 2015a). Therefore, in the present study, we tried to use the network pharmacology approach to explore predictive targets and therapeutic mechanisms underlying the action of EGCG against SC. We further verified the pharmacological effects of EGCG and the targets screened by network pharmacology by constructing a mouse model of septic cardiomyopathy.

## Methods

### Screening of molecular targets of EGCG against septic cardiomyopathy

**Figure 1:**
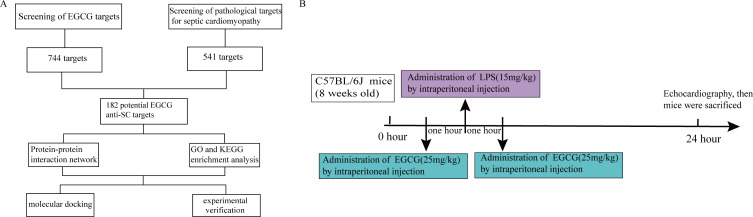
Flow chart of the analysis process in this study.

The plan of this study is shown in Fig. 1A. The pharmacological targets of EGCG were obtained by using the TCMID (Yu et al., 2021), TCMSP (Guan et al., 2021), STITCH (Ding et al., 2021), and SwissTargetPrediction (Li et al., 2021a) databases. Similarly, drugbank (Li et al., 2020a), Genecards (Zhang et al., 2021a), and OMIM (Liu et al., 2021) data were used to screen pathophysiological molecular targets for septic cardiomyopathy. A Venn diagram was used to evaluate the main targets of EGCG and septic cardiomyopathy to determine the potential targets of EGCG against septic cardiomyopathy.

### Construction of PPI network and screening of key genes

The potential therapeutic targets of EGCG for septic cardiomyopathy were analyzed by a PPI network, and the species were set to humans using the STRING database (http://string-db.org/) with a confidence level greater than 0.4 (Xiong et al., 2021). Then, the Cytoscape software (version 3.7.2) was used to visualize the PPI network.

To search the highly connected subnetworks in the PPI, Molecular Complex Detection (MCODE) of Cytoscape was used. A vertex-weighting-based scheme is used to identify local high-density areas in the graph, which is depicted in the MCODE results. The subnetwork with a cutoff MCODE score ≥ 10 was used for further analysis. The score value of a node reflects the density of this node and its surrounding nodes.

CytoHubba plug-in Cytoscape software was used to acquire the hub genes for EGCG anti-SC, which included 6 topological analysis methods: MCC (maximal clique centrality), degree, closeness, radiality, and stress (Chin et al., 2014). The degree of protein correlation in this module was scored by the following criteria: the degree cutoff was 2, and the node score cutoff was 0.2. These criteria were used to score the degree of protein correlation in this module, following node score = 0.2, degree = 2, K-score = 2 and max depth = 100 (Wang et al., 2020).

### GO and KEGG analysis of potential targets of EGCG Anti-SC

To comprehensively understand the potential target genes of EGCG in the treatment of septic cardiomyopathy, the DAVID database (https://david.ncifcrf.gov/) was used for GO and KEGG enrichment analysis (Ashburner et al., 2000; Kanehisa et al., 2017). *P* < 0.05 indicates that the entries and pathways are statistically significant. The ggplot2 package in R language was used to visualize the results.

### Docking analysis of targets and EGCG

To obtain their specific relationships, the mode between EGCG and the six potential target proteins were evaluated through molecular docking which predicts the extent of interactions. The 3D structure of the target protein was downloaded from the PDB database (http://www.rcsb.org/), and the 2D structure of EGCG was downloaded from the PubChem database (https://pubchem.ncbi.nlm.nih.gov/). Hetero and water molecules of the proteins were removed by PyMOL. AutoDock (version 4.2.6) was used to display the 3D grid box for molecular docking simulation. PyMOL and Discovery Studio 2020 were used to analyze the results.

### Verify the role of EGCG and the level of hub genes

#### Materials and animal treatment

Twenty-four male C57BL/6J mice (aged 8–10 weeks, weighing 22 ± 5 g) were purchased from Hunan SJA Laboratory Animal Co., Ltd., Changsha, China. EGCG (E4143, purity ≥ 95%) and lipopolysaccharide (LPS, 2880) were purchased from Sigma (St. Louis, MO, USA).

All animal treatments were carried out in accordance with the ARRIVE guidelines and Use Committee of the Second Affiliated Hospital of Nanchang University, China (NO. SYXK 2015-0001). All animals were housed in plexiglass maintained at 24 ± 3 °C and a relative 60 ± 10% of humidity. The food and water were sufficiently provided during the 12-hour light/dark cycle. After one week of adaptive feeding, they were random divided into three groups: the control group (*n* = 8), the LPS group (*n* = 8), and the LPS+EGCG group (*n* = 8). The three groups of mice were treated in the following ways: (1) the control group was administered an intraperitoneal injection of normal saline; (2) the mice in the LPS group were administered an intraperitoneal injection of LPS (15 mg/kg); and (3) in the LPS + EGCG group, all mice were injected with EGCG (25 mg/kg) before and 1 h after receiving an intraperitoneal injection of LPS (Fig. 1B). All mice were anesthetized with pentobarbital sodium (50 mg/kg) and sacrificed by cervical dislocation. The experiment was suspended and the mice were euthanized.

#### Echocardiography

The changes in cardiac function in all mice were evaluated by echocardiography 24 h after intraperitoneal injection of LPS. We used Vevo770 (VisualSonics, Toronto, Canada) with a 30 Hz transducer for transthoracic echocardiography. A short-axis view under M-mode tracings was used to measure the systolic and diastolic sizes of the left ventricle. The formulas for calculating ejection fraction ((EF)) and minor axis shortening ((FS)) are as follows: EF (%) = [(LVIDd)3 − (LVIDs)3/(LVIDd)3] × 100%. LV fractional shortening (FS) was calculated as [(LVIDd − LVIDs)/LVIDd] × 100%.

#### Histological examination

Part of the mouse heart tissue was immersed in 4% paraformaldehyde solution for 48 h and dehydrated step by step in ethanol. The preparation process was routinely stained with hematoxylin and eosin and finally sealed with paraffin.

#### Quantitative reverse transcription-PCR analysis

Total RNA was extracted from heart tissue by TRIzol (Beyotime, R0016). The purity and concentration of total RNA were detected by an instrument (NanoDrop™), and the absorbance of 260/280 RNA samples between 1.8 and 2.2 was used for the next step of reverse transcription. cDNA was synthesized according to the instructions of the reverse transcription kit (Tiangen, KR116). Real-time fluorescence PCR was used to detect the expression of mRNA in heart tissue by the SYBR Green method (Tiangen, FP313). The primer sequences for the genes used for RT-PCR detection are as follows (Table 1).

**Table 1:** Primers sequences of hub genes.

Gene	Primer nucleotide sequence
IL-6	Forward: 5′-CTGGTCTTCTGGAGTTCCGTTTCTAC-3′
	Reverse: 5′-GATGAGTTGGATGGTCTTGGTCCTTAG-3′
TNF	Forward: 5′-CCACGCTCTTCTGTCTACTGAACTTC-3′
	Reverse: 5′-GGTATGAAATGGCAAATCGGCTGAC-3′
MAPK3	Forward: 5′ATAGGCATCCGAGACATCCTCAGAG-3′
	Reverse: 5′-TTAAGGTCGCAGGTGGTGTTGATAAG-3′
VEGFA	Forward: 5′GGAGGAAGAGAAGGAAGAGGAGAGG-3′
	Reverse: 5′-CATGGTGGAGGTACAGCAGTAAAGC-3′
AKT1	Forward: 5′AGAGGCAGGAAGAAGAGACGATGG-3′
	Reverse: 5′-GCAGGACACGGTTCTCAGTAAGC-3′
Caspase3	Forward: 5′CACTGGAATGTCATCTCGCTCTGG-3′
	Reverse: 5′-GTCGCCTCTGAAGAAGCTAGTCAAC-3′

### Statistical analysis

All data are presented as means ± standard deviations (SD). GraphPad Prism 8.0.2 software (GraphPad Software Inc., San Diego, CA, U.S.) was utilized to conduct the statistical analyses. We used Student’s *t*-test for comparison of two groups and one-way ANOVA test with post hoc contrasts by Student-Newman-Keuls test was used to evaluate differences between two or more groups. *P* values <0.05 were considered statistically significant.

## Result

### Molecular formula and potential EGCG anti-SC molecular targets

**Figure 2:**
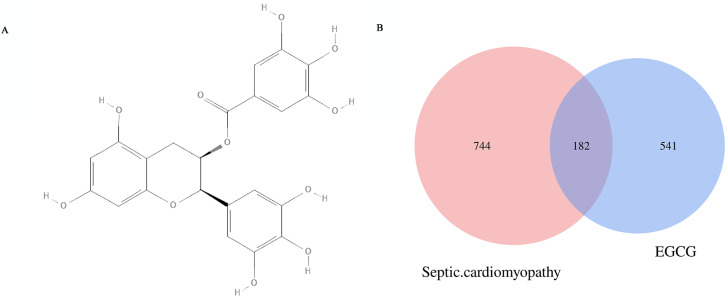
(A) Molecular structure of EGCG. and potential targets. (B) The Venn diagram represen 541 EGCG targets and intersects 744 key genes of septic cardiomyopathy to obtain 182 potential targets. (C) The blue diamond represents the key gene of septic cardiomyopathy, the green octagon represents the target of EGCG, and the red square represents the potential target of EGCG in the treatment of septic cardiomyopathy.

The chemical formula of EGCG (Fig. 2A) was obtained from the PubChem database. The CAS number is 989-51-5. A total of 744 genes (after removing duplicates) related to septic cardiomyopathy were obtained from the DrugBank, GenBank and OMIM databases. Five hundred forty-one EGCG drug targets were obtained through the TCMID, TCMSP, SwissTargetPrediction and Stitch databases. The 182 potential targets of EGCG against septic cardiomyopathy were shown by using Venn diagrams (Fig. 2B).

### Acquisition results of hub genes for EGCG anti-SC and PPI network construction

**Figure 3:**
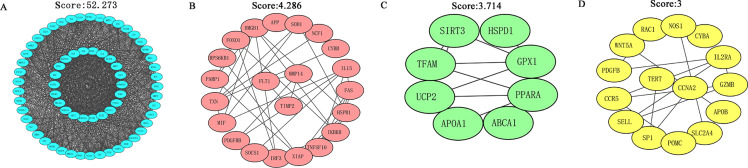
MCODE in Cytoscape software was used to analyze the PPI network and four functional module clusters were obtained according to different scores.

The STRING database was applied to construct a function-related PPI network from the 182 targets by using a minimum required interaction score set to 0.4. First, we set the species to “Homo sapiens” and then entered 182 potential therapeutic targets for EGCG anti-SC to obtain the PPI network (Fig. S1). Next, we imported the TSV files into Cytoscape software (version 3.7.2) for further analysis and visualization. Cytoscape software was used to analyze the PPI network based on the MCODE topology, and similar functional clusters were selected to find closely connected areas. Four functional module clusters were detected according to the score, as shown in Fig. 3. Gene clusters with a score equal to 52.273 were used for further analysis.

**Figure 4:**
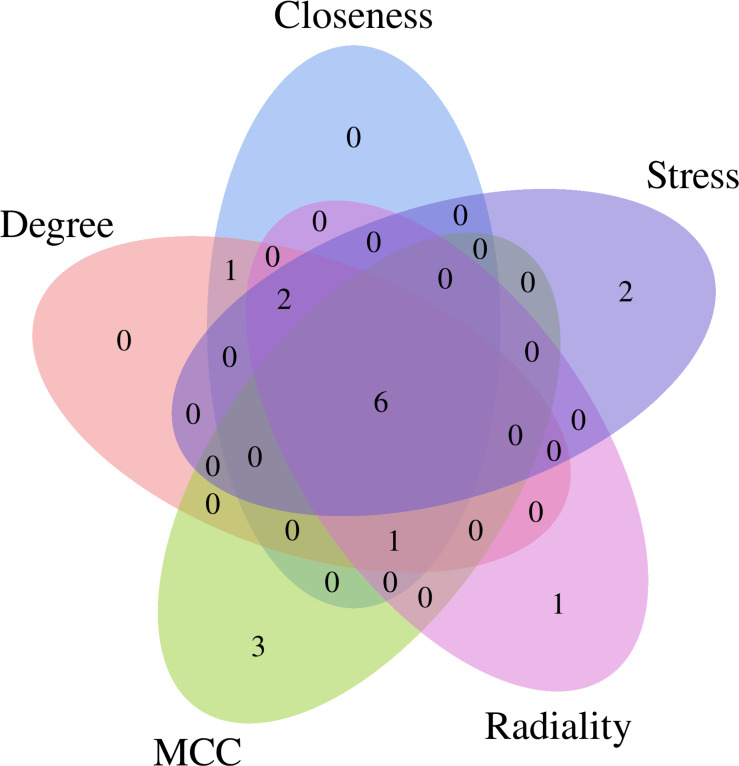
Five topological analysis methods of CytoHubba plug-in of Cytoscape software were used to analyze the gene clusters with a score of 52.273, and six key targets of EGCG in the treatment of septic cardiomyopathy were obtained.

The CytoHubba plug-in of Cytoscape software was used to obtain the hub genes for EGCG anti-SC based on the above cluster (score = 52.273), which currently contains 5 topological analysis methods (Fig. 4). The top 10 hub genes for EGCG anti-SC were obtained, and the results of 12 algorithms included 10 genes each. Finally, the hub genes included IL-6, TNF, Akt1, VEGFA, MAPK3, and Casp3, which were acquired by intersecting the genes obtained by the five algorithms (Fig. S2).

### GO and KEGG pathway enrichment analysis

**Figure 5:**
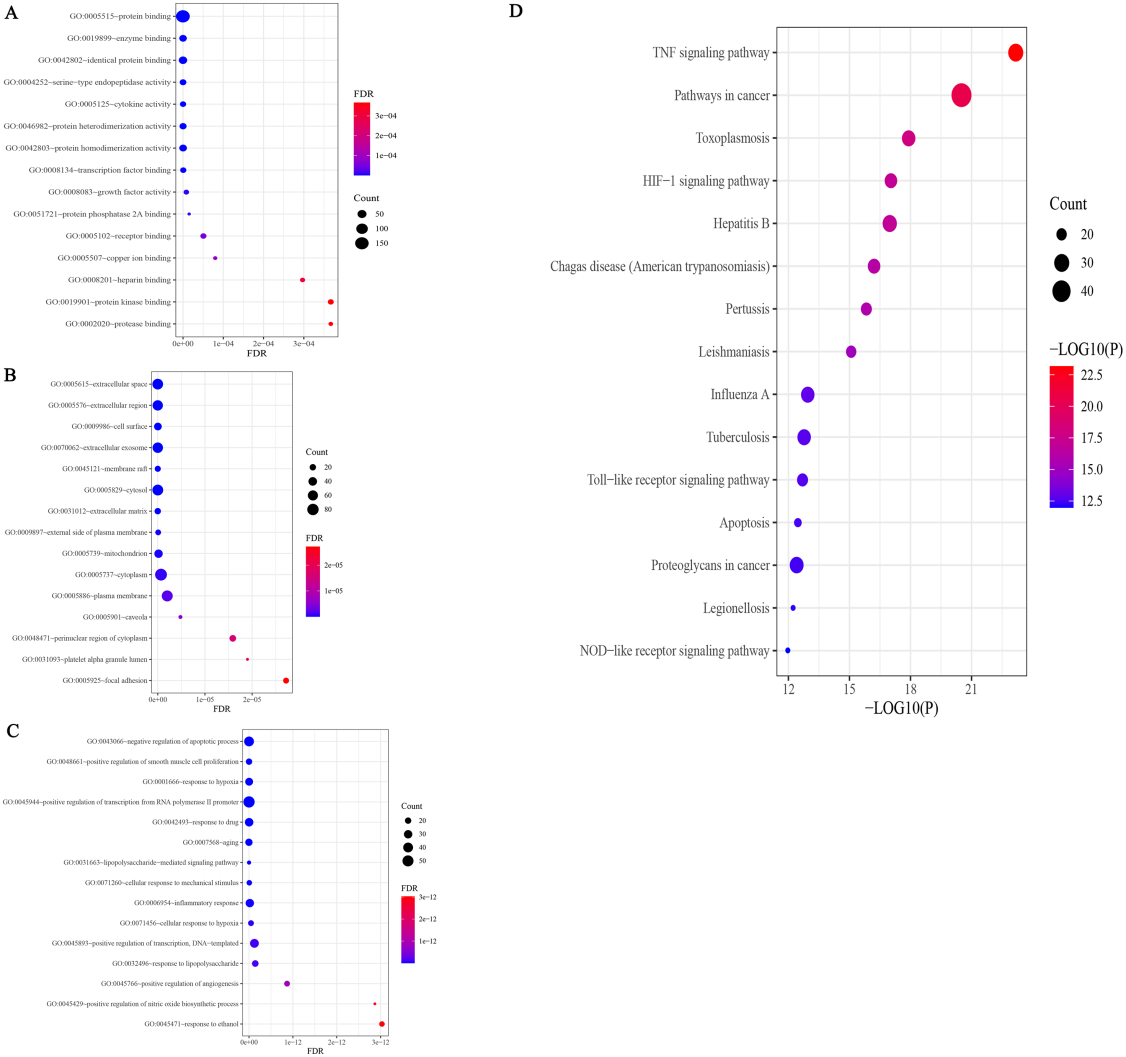
GO enrichment analysis of potential targets.

The results of GO analysis showed that there were 943 terms, including 750 terms (Table S1) for biological process (BP), 78 terms for cellular component (CC), and 115 terms for molecular function (MF). The results of each terms were sorted by the number of genes and significance, and the top 15 findings of each analysis were selected for display (Fig. 5A). Among them, 750 BP terms included positive and negative regulation of apoptosis, inflammatory response, response to lipopolysaccharide, *etc*.; 78 CC terms included cytoplasm, plasma membrane, nucleus, and mitochondria, and 115 MF terms included protein binding, identical protein binding, enzyme binding, ATP binding, cytokine activity, and transcription factor binding, *etc*. The results of GO enrichment analysis of SC treated with EGCG showed that its biological process was mainly reflected in the regulation of apoptosis, signal transduction, immune response, and lipopolysaccharide response; the molecular function was mainly reflected in protein binding, cytokine activity, transcription factor activity, and ATP binding; and the composition was mainly reflected in the cytoplasm, plasma membrane, and nucleus. *P* < 0.05 was the filtering parameter of the cutoff point, and 119 KEGG pathway items were acquired (Table S2). The first 15 records filtered from small to large *P* values are shown in Fig. 5A. KEGG enrichment analysis involves infectious diseases caused by many pathogens, including the TNF signaling pathway, the HIF-1 signaling pathway, the Toll-like receptor signaling pathway, the apoptosis signaling pathway, the PI3K/AKT signaling pathway, and the NF-*κ*B signaling pathway (Fig. 5B).

### Molecular docking results

Molecular docking is used to verify the binding affinity between EGCG and protein targets. The docking score indicates the binding affinity. Thus, the lowest score signifies the highest binding affinity between EGCG and the target protein. From this, the correct binding posture has been selected to analyze the interactions between EGCG and its target.The hub protein targets are filtered by the top 5 node degrees of the PPI network and seed nodes of clusters, including IL-6, TNF, MAPK3 (Erk1/2), VEGFA, AKT1, and CASP3. The results of the molecular docking of drugs and target proteins are shown in Table 2. EGCG is compared with each target under the condition that all target proteins met physiological pH (pH = 7.35). The grid box is located in the center, covering the active binding site and all necessary residues. For IL6, the grid box (100 Å × 100 Å × 100 Å) is centered at (30.183, 58.501, 20.278) Å; for TNF, the grid box (92 Å × 92 Å × 92 Å) is centered at (−33.22, 41.788, 39.681) Å; for MAPK3, the grid box (126 Å × 126 Å × 126 Å) is centered at (57.761, 4.848, −3.43) Å; for VEGFA, the grid box (40 Å × 40 Å × 40 Å) is centered at (10.383, −18.002, 25.541) Å; for AKT1, the grid box (118 Å ×106 Å × 86 Å) center at (21.763, 14.444, 9) Å; for the CASP3, grid box (60 Å × 60 Å × 60 Å) center at (24.892, 53.581, 12.294) Å. At present, there is no unified standard to screen active molecules. According to the literature (Guan et al., 2021), we select the active components with binding energy ≤ −5.0 kJ/mol as the basis for screening.It can be seen from Table 2 that the binding affinity of EGCG to the crystal structure of 6 core proteins is lower than -5 kcal/mol, indicating that EGCG exhibits notable binding affinity for the native structure of the target proteins. EGCG show the high binding affinity of −5.73 kcal/mol in IL6, −7.86 kcal/mol in TNF, −6.81 kcal/mol in MAPK3, −5.45 kcal/mol in VEGFA, −6.26 kcal/mol in AKT1, and −7.07 kcal/mol in CASP3, and the exhaustive value of all molecular docking results is 6. The results show that CASP3 is the most effective target for sepsis cardiomyopathy, both in terms of binding energy and the number of hydrogen bonds.

**Table 2:** The binding energy values of EGCG and core targets.

Compund	Targets	Binding affinity/ (kcal/mol)
EGCG	IL-6	−5.73
EGCG	TNF	−7.86
EGCG	MAPK3	−6.81
EGCG	VEGFA	−5.45
EGCG	AKT1	−6.26
EGCG	Caspase3	−7.07

**Figure 6:**
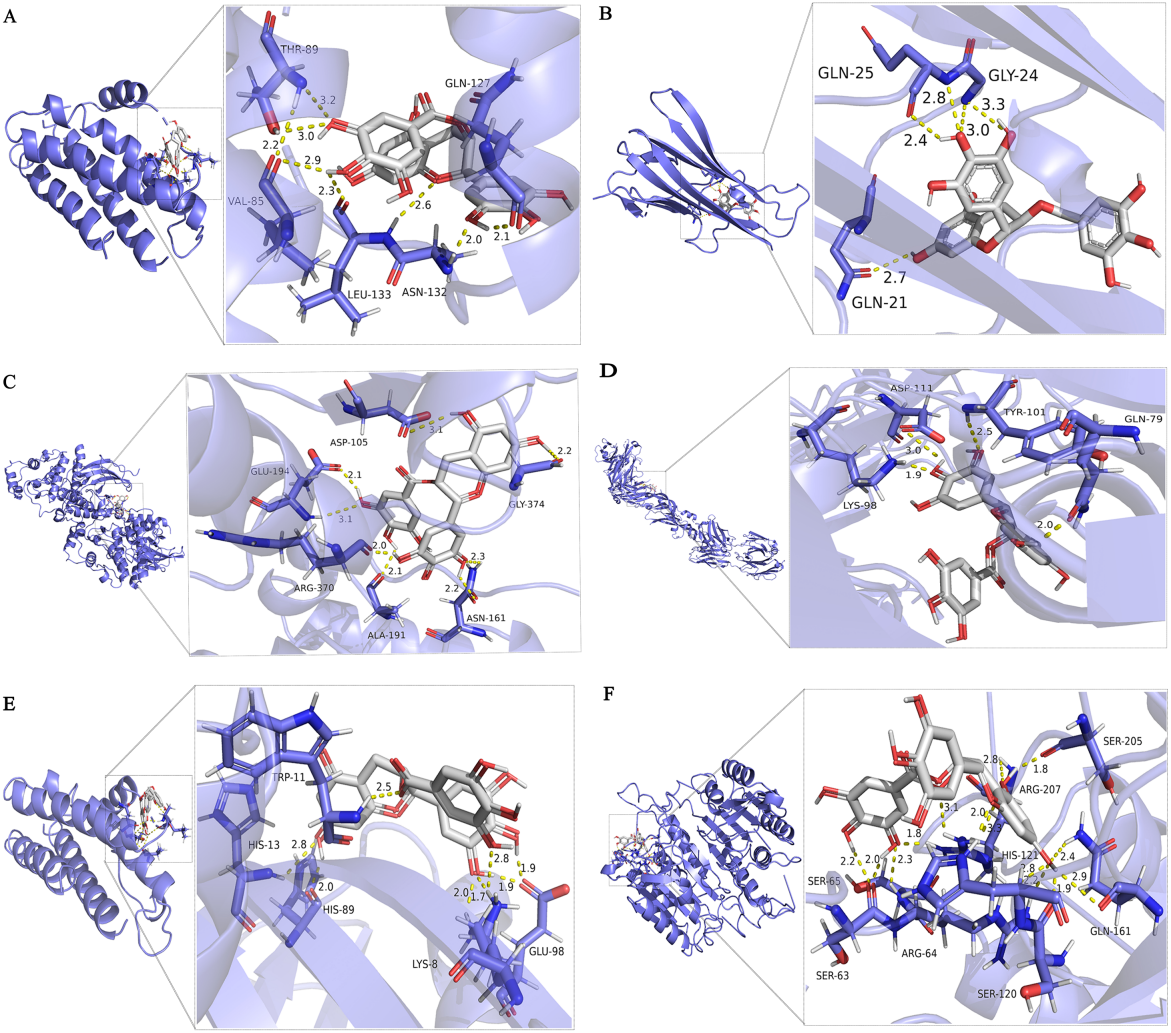
Molecular docking simulation of EGCG-target binding.

After docking we have obtained six docked poses in each case or for each protein target. According to the number and distance of hydrogen bond forces (distance ≤ 3.0 Å), it is concluded that the tertiary structure 3h0e of CASP3 is the best structure for molecular docking. Small molecular compounds bind closely to protein residues through various interactions (Fig. 6).

### Effect of EGCG on functional and morphological changes of echocardiography induced by LPS

**Figure 7:**
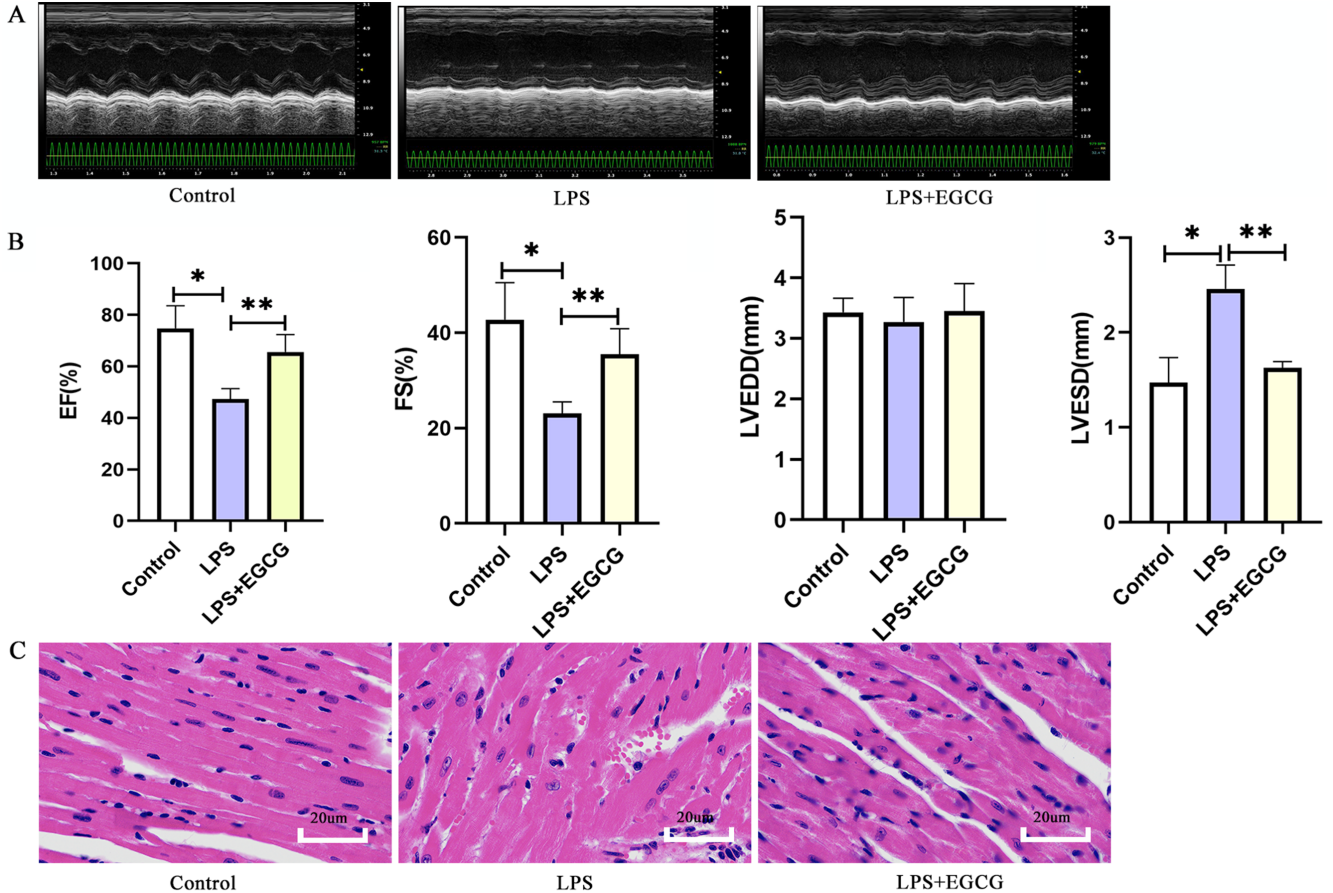
Treatment of EGCG ameliorated cardiac dysfunction and histopathological injury in heart induced by LPS.

The echocardiography results in mice showed that LPS significantly increased the left ventricular end-systolic diameter (LVESD), decreased the ejection fraction and shortening fraction and had little effect on the left ventricular end-diastolic diameter (LVEDD) (Fig. 7B). In addition, EF and FS in LPS + EGCG group were significantly higher than those in LPS group. It is suggested that EGCG can improve the cardiac function of mice induced by LPS. The morphological changes of the myocardium were evaluated by HE staining. As shown in the figure (Fig. 7C), compared with the control group, the arrangement of cardiomyocytes in the LPS group was more disordered, with interstitial edema, some infiltration of inflammatory cells and a small amount of exudation of red blood cells that could be seen in the interstitium. Compared with the LPS group, the cardiomyocytes in the LPS+EGCG group were neatly arranged, interstitial edema was alleviated, and there was no obvious inflammatory cell infiltration or erythrocyte exudation.

### Experimental verification of target genes expression in LPS-induced mouse model

**Figure 8:**
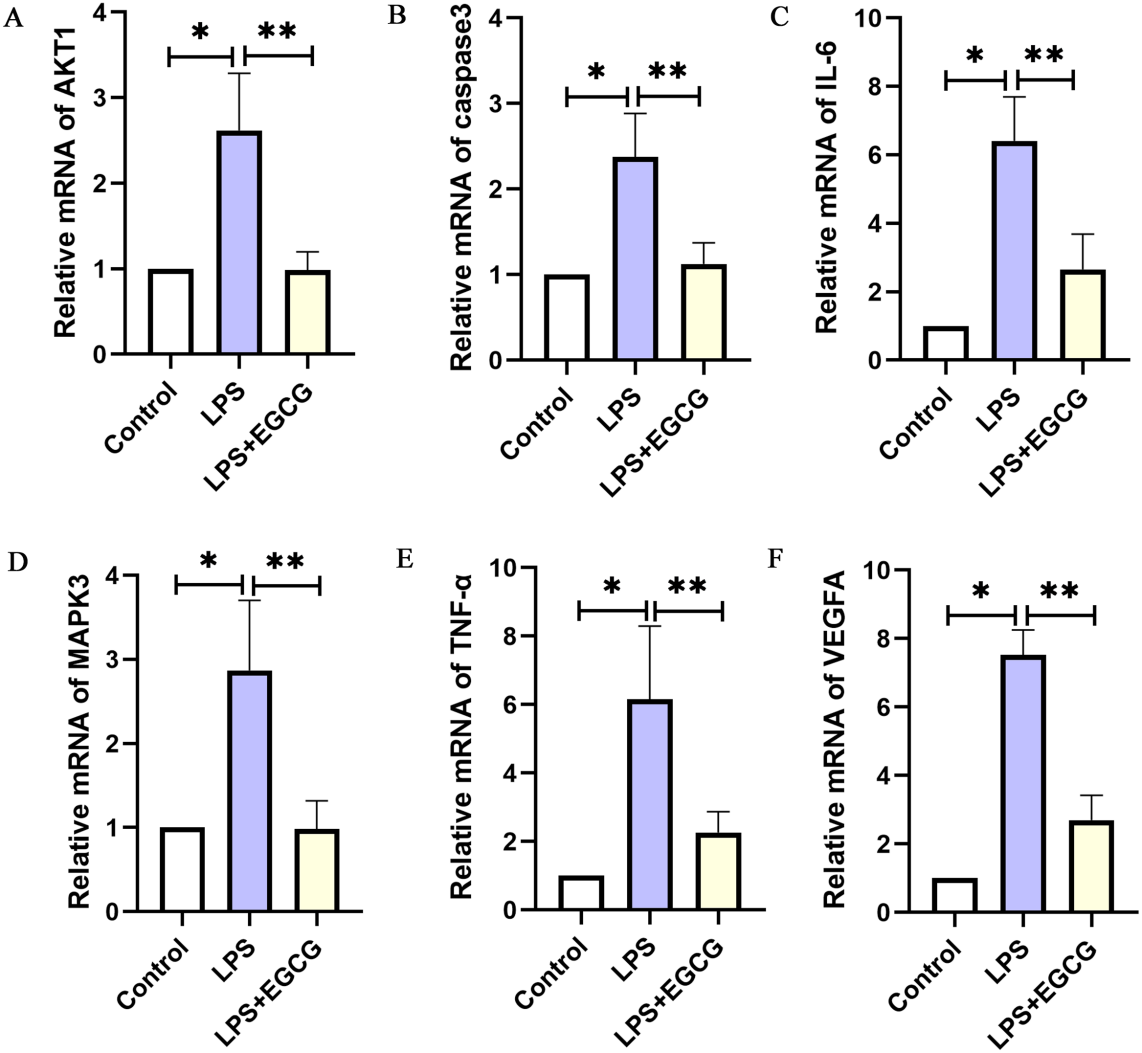
The mRNA expression of the key genes of EGCG in the treatment of septic cardiomyopathy in each group of mice.

As shown in Fig. 8, the RT-PCR results showed that the expression of key genes (IL-6, TNF-a, Caspase-3, VEGFA, Akt1, MAPK3) in the LPS group was higher than in the control group. Compared with that in the LPS group, the expression of key genes (IL-6, TNF-a, caspase-3, VEGFA, Akt1, MAPK3) in the LPS+EGCG treatment group was decreased. From the Genecards database, IL-6, TNF-α, MAPK3 and VEGFA are related to inflammation, while Caspase-3 and AKT1 are related to apoptosis. Therefore, EGCG can inhibit inflammation and apoptosis.

## Discussion

Septic cardiomyopathy is a common complication of sepsis, with an incidence of 10–70% in patients with sepsis (Zhang et al., 2021b). Septic cardiomyopathy is often characterized by systolic or diastolic dysfunction, which causes further ischemia and hypoxia in peripheral tissue, aggravates the dysfunction of other organs, and leads to increased mortality (Martin et al., 2019). Therefore, it is necessary to identify an effective drug for the treatment of septic cardiomyopathy. EGCG is a polyphenol component of green tea that has anti-inflammatory, antioxidant, antiviral and antitumor effects (Eng, Thanikachalam & Ramamurthy, 2018). Some studies have shown that EGCG plays an important role in preventing cardiovascular disease, participating in antiplatelet aggregation (Joo et al., 2018; Lee et al., 2013), decreasing atherosclerosis (Duan et al., 2020; Yamagata, 2020), counteracting antiventricular remodeling (Cai et al., 2021; Ma et al., 2019) and regulating blood lipids (Ma et al., 2019; Liu et al., 2013). Network pharmacology is used to study the intervention mechanism of drugs on diseases through the construction of biological interaction networks, which systematically reveal the therapeutic effects of drugs on diseases from the interaction of drugs, targets and diseases (Kibble et al., 2015b). In previous studies, EGCG improved intestinal injury (Ma et al., 2021), cerebral nervous system injury (Cheng et al., 2021), lung injury (Wang, Fan & Zhang, 2019) and endothelial injury (Baek et al., 2019) in LPS-induced sepsis in mice. Li et al. (2020b) found that EGCG can inhibit AKT phosphorylation and the ERK signaling pathway to improve LPS-induced H9C2 cell injury. As shown in Fig. 7, EGCG improved cardiac function and myocardial injury in LPS-induced mice. Therefore, we conclude that EGCG may be involved in exerting a protective effect in septic cardiomyopathy.

While the pathogenesis of sepsis is not completely clear, it is mainly related to the release of circulating myocardial inhibitory factors, downregulation of sarcomere- and mitochondrial-related genes, downregulation of the adrenergic pathway, changes in coronary microcirculation, activation of the inflammatory response, mitochondrial dysfunction, increased oxidative stress and abnormal calcium regulation (Hollenberg & Singer, 2021). Inflammation, as the initiator of septic cardiomyopathy, promotes an increase in intracellular oxidative stress, which can cause cardiomyocyte apoptosis and lead to cardiac dysfunction. The anti-inflammatory effect of EGCG has been verified in many studies. EGCG ameliorates cigarette smoke-induced myocardial inflammation through MAPK and NF-κB (Liang, Ip & Mak, 2019). After administration of EGCG for one month, the level of serum proinflammatory factors (IL-1β, TNF-α, IL-6) in streptozotocin-induced diabetic mice decreased (Othman et al., 2017a).

The results of GO enrichment analysis further confirmed that the candidate target protein of EGCG is mainly involved in the process of inflammation and apoptosis. In our results, BP enrichment of anti-inflammatory factors included an inflammatory response, signal transduction, response to lipopolysaccharide, and cell adhesion, suggesting that the anti-inflammatory effects of EGCG range from inflammatory cell adhesion and inhibition of the cell response to lipopolysaccharide and intracellular signal transduction. In addition to the anti-inflammatory process, the enrichment of BP also includes apoptosis and oxidation–reduction processes, which is consistent with the pathogenesis of septic cardiomyopathy, indicating that the protective effect of EGCG on septic cardiomyopathy is multifaceted.

Six targets with a high degree of PPI network (IL-6, TNF, MAPK3, VEGFA, AKT1 and Caspase3) are identified according to the analysis of compound-target interactions. In the molecular docking analysis of our study, the binding mode with the highest docking fraction is chosen to analyze the interaction between EGCG and protein receptors. These six targets of EGCG in the treatment of septic cardiomyopathy are screened out by network pharmacology, and they are mainly related to inflammation and apoptosis. This finding is consistent with the results of GO enrichment. IL-6 and TNF are common markers of inflammation. EGCG protects against LPS-induced lung injury by promoting PRCKA and inhibiting the expression of IL-6 and TNF (Wang et al., 2021). EGCG protects the myocardium of streptozotocin-nicotinamide-induced diabetic rats through anti-inflammatory and anti-apoptotic pathways, mainly through diminishing the levels of IL-6, TNF-α and Caspase3 (Othman et al., 2017a). In addition, EGCG can inhibit cardiomyocyte apoptosis induced by isoproterenol by reducing the level of Caspase3 (Othman et al., 2017b). MAPK3, also known as ERK1, is involved in inflammation (Yang et al., 2019), proliferation (Mebratu & Tesfaigzi, 2009) and apoptosis (Cook et al., 2017). AKT1, also known as protein kinase B, is one of the three subunits of the AKT family and is involved in cell proliferation, inflammation, survival, metabolism and angiogenesis. Many studies have suggested that Akt1 has a protective effect in cardiovascular disease, but some studies still disagree. AKT1 deficiency can prolong the life span in the DKO mouse model, improve cardiac function and reduce myocardial hypertrophy (Kerr et al., 2013). Akt1 increases oxidative stress in DKO cells and promotes cell senescence and apoptosis by inhibiting the expression of ROS scavengers downstream of FoxO (Nogueira et al., 2008). The Akt1/NF-κB axis is involved in the inflammatory injury process of collagen-induced arthritis in mice  (Yang et al., 2020). The docking scores of EGCG targets are all less than -5 kcal/mol, indicating that EGCG has a good binding affinity for all six targets. Table 2 shows that the binding energy of EGCG and AKT1 is 6.26 kcal/mol, which shows that EGCG can combine well with Akt1. These results suggest that EGCG may be involved in inhibiting the AKT1/NF-κB axis and improving myocardial injury in septic cardiomyopathy. VEGFA is a member of the PDGF/VEGF growth factor family. It is essential for physiological and pathological angiogenesis to induce the proliferation and migration of vascular endothelial cells. Serum VEGFA in peritoneal dialysis patients is proportional to inflammatory factors such as IL-6. However, miR-15a-5p suppresses the inflammatory response of human peritoneal interstitial cells by targeting VEGFA (Shang et al., 2018). VEGFA promotes cardiomyocyte apoptosis induced by hypoxia, and miR-448-5p targeting VEGFA can inhibit the expression of Caspase3 (Tang et al., 2021). The binding energy of EGCG and VEGFA is 5.45 kcal/mol. EGCG combined with VEGFA may be involved in the inhibition of inflammation and apoptosis. Caspase3 is a downstream protein of the apoptotic pathway and participates in the progression of septic cardiomyopathy (Cao et al., 2020). Sulfur dioxide reduces Caspase3, inhibits cardiomyocyte apoptosis and improves myocardial injury in mice with CLP (Yang, Zhang & Chen, 2018). Therefore, we believe that the myocardial protective effects of EGCG may be associated with the regulation of caspase-3.

**Figure 9:**
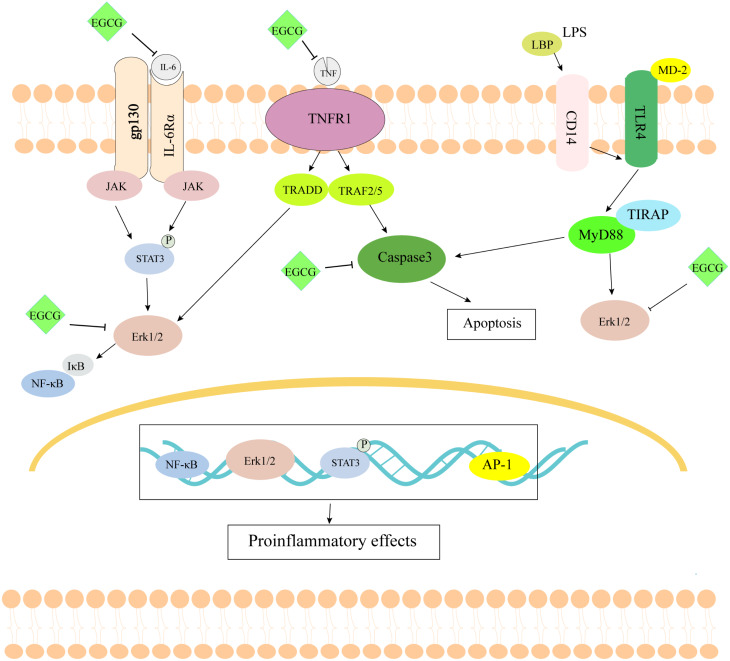
The schematic diagram of EGCG in the treatment of septic cardiomyopathy.

## Conclusion and Limitation

As shown in Fig. 9, EGCG probably improves myocardial injury in sepsis through anti-inflammatory and anti-apoptotic effects. Through the study of network pharmacology, it is concluded that EGCG can not only bind directly to inflammation-related proteins to inhibit inflammation, but it also binds to apoptosis-related proteins to inhibit apoptosis. Through the construction of the LPS-induced sepsis model to verify the key targets, it can be concluded that EGCG can reduce the expression of inflammation- and apoptosis-related genes. However, there are still some limitations to this study. First, we screened the molecular targets of human septic cardiomyopathy in the database, but we verified it using a mouse model. Second, through the screening of network pharmacology, we are bound to miss the important target of EGCG. Finally, we only verified the effect of EGCG on the target at the mRNA level, not the protein level of the target. In summary, our findings may provide a solution for the treatment of septic cardiomyopathy.

## Supplemental Information

10.7717/peerj.12994/supp-1 Supplemental Information 1 ARRIVE Checklist

10.7717/peerj.12994/supp-2 Supplemental Information 2 The PPI network diagram and the results of GO and KEGG enrichment analysis

## Additional Information and Declarations

### Data Availability

The following information was supplied regarding data availability: The raw measurements are available in the Supplementary Files.
