# Supplementary figures and images for: Analysis of the role and mechanism of EGCG in septic cardiomyopathy based on network pharmacology
Source: PeerJ. 2022 Mar 9;10:e12994. doi: 10.7717/peerj.12994 (PMC8917800; doi:10.7717/peerj.12994)

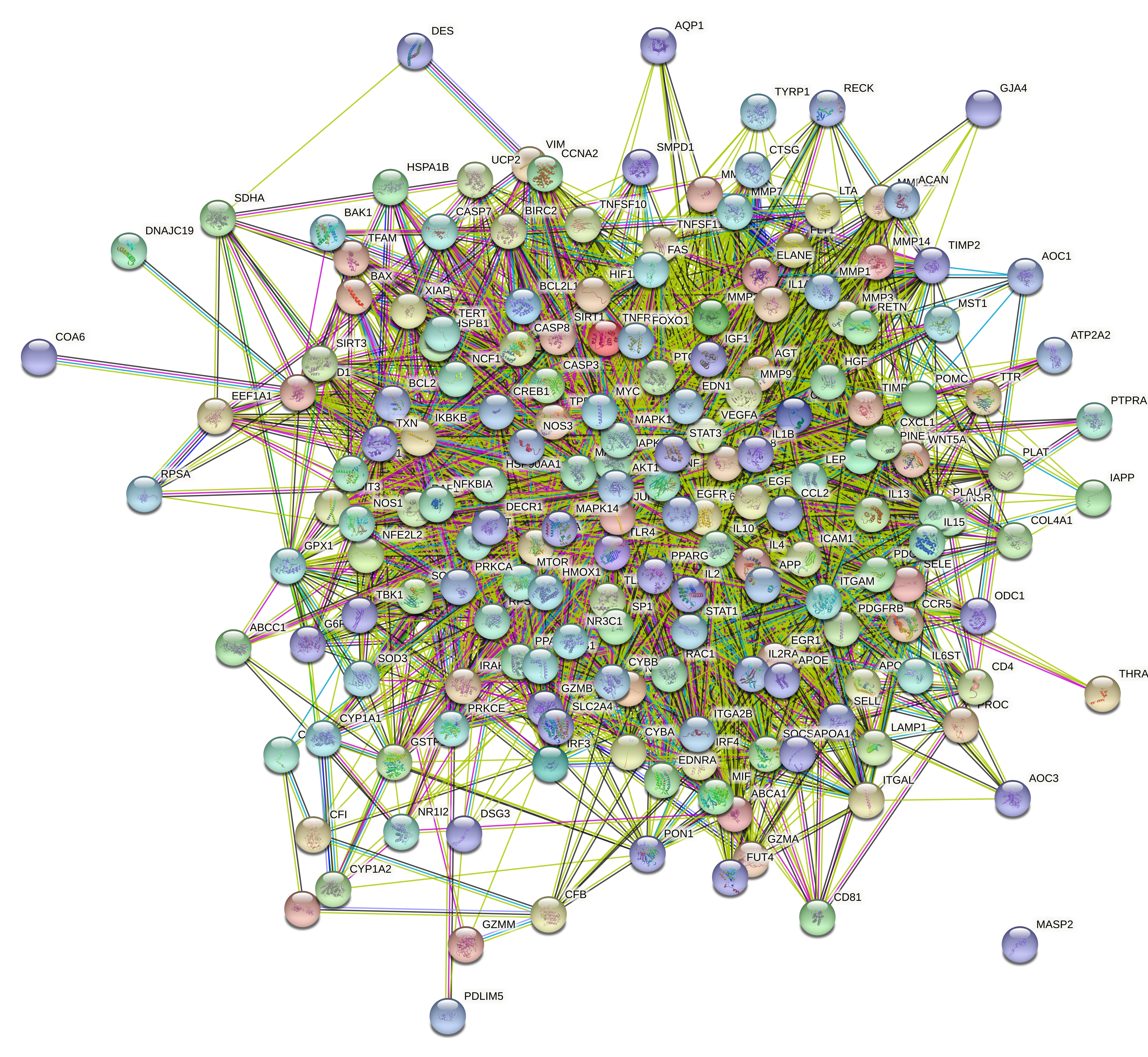

Supplement: Supplemental Information 2 [file peerj-10-12994-s002.zip › Supp Figs and Tables 63721 JT staff/Supplementary file/FigS1.png]
